# Identification of Anion Channels Responsible for Fluoride Resistance in Oral Streptococci

**DOI:** 10.1371/journal.pone.0165900

**Published:** 2016-11-08

**Authors:** Xiaochen Men, Yukie Shibata, Toru Takeshita, Yoshihisa Yamashita

**Affiliations:** Section of Preventive and Public Health Dentistry, Division of Oral Health, Growth and Development, Faculty of Dental Science, Kyushu University, Fukuoka, Japan; LSU Health Sciences Center School of Dentistry, UNITED STATES

## Abstract

Recently, it has been reported that *eriC* and *crcB* are involved in bacterial fluoride resistance. However, the fluoride-resistance mechanism in oral streptococci remains unclear. BLAST studies showed that two types of *eriC*s (*eriC1* and *eriC2*) and two types of *crcB*s (*crcB1* and *crcB2*) are present across 18 oral streptococci, which were identified in ≥ 10% of 166 orally healthy subjects with ≥ 0.01% of the mean relative abundance. They were divided into three groups based on the distribution of these four genes: group I, only *eriC1*; group II, *eriC1* and *eriC2*; and group III, *eriC2*, *crcB1*, and *crcB2*. Group I consisted of *Streptococcus mutans*, in which one of the two *eriC1*s predominantly affected fluoride resistance. Group II consisted of eight species, and *eriC1* was responsible for fluoride resistance, but *eriC2* was not, in *Streptococcus anginosus* as a representative species. Group III consisted of nine species, and both *crcB1* and *crcB2* were crucial for fluoride resistance, but *eriC2* was not, in *Streptococcus sanguinis* as a representative species. Based on these results, either EriC1 or CrcBs play a role in fluoride resistance in oral streptococci. Complementation between *S*. *mutans* EriC1 and *S*. *sanguinis* CrcB1/CrcB2 was confirmed in both *S*. *mutans* and *S*. *sanguinis*. However, neither transfer of *S*. *sanguinis* CrcB1/CrcB2 into wild-type *S*. *mutans* nor *S*. *mutans* EriC1 into wild-type *S*. *sanguinis* increased the fluoride resistance of the wild-type strain. Co-existence of different F^−^ channels (EriC and CrcB) did not cause the additive effect on fluoride resistance in oral *Streptococcus* species.

## Introduction

Fluoride is commonly used as an effective caries-preventive agent in many countries. Organisms present in the oral cavity are frequently exposed to fluoride ions from drinking water or from the use of fluoride dentifrices and fluoride mouth rinses. Fluoride is known to interfere with metabolic processes in many organisms [1], and high-concentration fluoride shows a bactericidal effect [2, 3]. These anti-microbial effects contribute to the anti-caries effect of fluoride. However, the anti-microbial effects of fluoride may have an unexpected effect on the bacterial composition of oral microflora.

The widespread, long-term use of fluoride can result in the emergence of fluoride-resistant oral *Streptococcus* species, including *Streptococcus mutans*, a cariogenic bacterium [4–6]. These fluoride-resistant strains show clear phenotypic differences in growth, adherence, and metabolic activity compared to the fluoride-sensitive strains [7–9]. The emergence of fluoride-resistant oral *Streptococcus* species may not only decrease the anti-caries effects of fluoride, but also disrupt the composition of oral streptococci in the oral cavity, followed by the deterioration of oral health.

More than 700 bacterial species are present in the oral cavity, and oral streptococci are predominant. Oral streptococci account for approximately 20% of all bacteria in saliva [10] and approximately 50% of those during the early stages of dental plaque formation [11]. Oral streptococci pioneer early dental plaque formation and have a specific temporal and spatial distribution that is crucial for the development of oral biofilms [12].

Bacteria have evolved numerous strategies to alleviate the toxic effects of ions other than fluoride, and yet analogous systems for fluoride toxicity mitigation were notably absent [13]. Recently, it has been reported that the *eriC* gene of *Pseudomonas syringae* and the *crcB* gene of *Escherichia coli* are involved in bacterial fluoride resistance [14]. The *eriC* gene is described as a ClC chloride channel in many bacteria, and the *crcB* gene has previously been implicated in resistance to camphor-induced chromosome decondensation. Furthermore, Liao *et al*. [15] identified two single nucleotide polymorphisms (SNPs) in the gene cluster, including two *eriC* genes in the genome of the fluoride-resistant mutant *S*. *mutans* C180-2FR. Expression of the cluster was approximately 10-fold higher in C180-2FR than in the parent strain C180-2. These results suggested that *eriC* may be related to the response to fluoride in *S*. *mutans*. However, it remains unclear whether *eriC* and/or *crcB* play a role in fluoride resistance in oral streptococci. To maintain the appropriate bacterial composition of oral microflora, it is important to investigate the fluoride resistance mechanism in oral streptococci. In this study, we attempted to identify and characterize fluoride-resistance-related genes in oral streptococci.

## Materials and Methods

### Bacterial strains and culture conditions

The following bacterial strains were used in this study: *Streptococcus mutans* UA159, *Streptococcus sobrinus* OMZ175, *Streptococcus mitis* ATCC 49456, *Streptococcus oralis* ATCC 10557, *Streptococcus gordonii* ATCC 10558, *Streptococcus sanguinis* SK36, *Streptococcus parasanguinis* ATCC 15912, *Streptococcus tigurinus* ATCC 15914, *Streptococcus australis* ATCC 700641, *Streptococcus infantis* ATCC 700779, *Streptococcus salivarius* HHT, *Streptococcus anginosus* NCTC 10707, *Streptococcus intermedius* ATCC 27335, and *Escherichia coli* DH5α

*E*. *coli* strains were grown in 2×YT Broth (Becton Dickinson, Franklin Lakes, NJ, USA). Oral streptococci strains were grown in brain heart infusion (BHI) broth (Becton Dickinson) at 37°C in 5% CO_2_. Antibiotics were used at the following concentrations: 300 μg/mL erythromycin and 50 μg/mL ampicillin for *E*. *coli*, 20 μg/mL erythromycin for oral streptococci, 600 μg/mL spectinomycin for *S*. *mutans* and *S*. *anginosus*, and 150 μg/mL spectinomycin for *S*. *sanguinis*.

### DNA Manipulation

Standard recombinant DNA procedures such as DNA isolation, endonuclease restriction, ligation, and agarose gel electrophoresis were performed as described by Sambrook & Russell [16]. Transformations of oral streptococci and *E*. *coli* were performed as described previously [17, 18]. Protein sequence similarity searches were performed with the BLAST program via the National Center for Biotechnology Information server.

### Construction of mutant forms of the genes in *Streptococcus* species

Various deletion mutants were constructed by replacing the target gene with an erythromycin resistance (Em^r^) or spectinomycin resistance (Spc^r^) gene using double cross-over homologous recombination, as described previously [19]. As an example, we describe a strategy for construction of the *eriC1a*/*eriC1b* double mutant. An 869-bp fragment upstream of *eriC1a* and a 767-bp fragment downstream of *eriC1b* were amplified from *S*. *mutans* UA159 genomic DNA and inserted upstream and downstream, respectively, of the Em^r^ gene in pBSSKII-Em^r^ [20], in which the Em^r^ fragment was cloned into HindIII- and EcoRV-digested pBluescript SK II (+). The resultant plasmid (pBSSKII-Em^r^-*eriC1a*/*eriC1b* -UD) was digested with KpnI and SacII, and the assembled fragment was transformed into *S*. *mutans* UA159. Correct insertions or replacements of transformants were confirmed by PCR.

### Complementation between *Streptococcus* species

Complementation between *Streptococcus* species was performed using the shuttle vector, pSEP, which consisted of an Em^r^ gene, pC194ori for replication in Gram-positive organisms, pUCori for replication in *E*. *coli*, and the promoter region of the Em^r^ gene derived from Gram-positive organisms inserted within the multi-cloning site (MCS). A target gene was amplified by PCR from the parent strain genomic DNA and cloned downstream of the promoter region within the MCS. Prior to complementation, Em^r^ within plasmids for construction of the mutant strains were replaces with Spc^r^. Complementing plasmid was introduced into Spc^r^-mutant and transformants were selected on BHI agar plates containing appropriate concentration of erythromycin and spectinomycin. The existence of complementing plasmid was confirmed by plasmid extraction from transformants.

### Evaluation of the fluoride sensitivity of strains

The mutant strains and wild-type control strain cells were grown in BHI broth overnight at 37°C in 5% CO_2_. The cultures were then diluted 1:10 into fresh BHI and grown to an OD_550_ of ~0.5. Aliquots (20 μL) of cell suspensions with the same turbidity were inoculated into wells containing 200 μL of fresh BHI medium with several different sodium fluoride (NaF) concentrations. The ranges of NaF concentrations tested were selected based on preliminary experiments in which growth rates of oral streptococci were examined in dilution series of NaF using planktonic culture. Growth was evaluated after incubation for 16 h by measuring OD_550_ using a SpectraMax 340PC384 microplate spectrophotometer (Molecular Devices, Sunnyvale, CA, USA). Wells containing only BHI were used as controls. Growth yields were estimated based on the means of the data obtained from three independent experiments.

### Statistical analysis

Data were expressed as mean ± standard deviation (SD). The Bonferroni test was used to determine the significance of differences in multiple comparisons. Differences were considered significant only for values of *P* < 0.05. All statistical analyses were performed using SPSS for Windows version 22.0 (SPSS, Inc., Chicago, IL, USA).

## Results

### Distribution of *eriC* and *crcB* genes in oral streptococci

**Fig 1:**
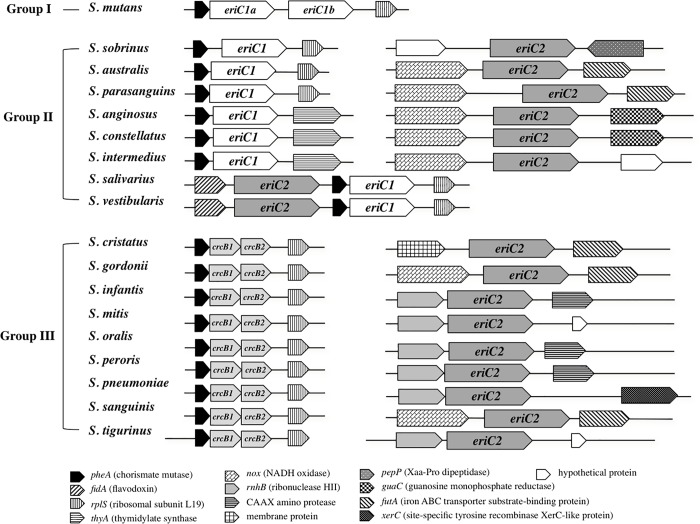
Locations of *eriC* and *crcB* genes in 18 oral streptococci. These species were selected based on the following criteria: identification in ≥ 10% of 166 orally healthy subjects and ≥ 0.01% of the mean relative abundance [10].

To examine the distribution of *eriC* and *crcB* genes in oral streptococci, we performed sequence homology analysis for the complete genome sequences of 18 types of oral streptococci. These species were selected based on the following criteria: identification in ≥ 10% of 166 orally healthy subjects and ≥ 0.01% of the mean relative abundance [10]. There were two types of *eriC* genes (*eriC1* and *eriC2*) and two types of *crcB* genes (*crcB1* and *crcB2*) in oral streptococci (Fig 1). The *eriC1* gene product showed about 50% similarity with EriC of *P*. *syringae* DC3000, which was identified as a fluoride channel protein in a previous report [14]. On the other hand, another EriC2 had no significant similarity with *P*. *syringae* EriC. Both *crcB* products showed about 50% similarity with CrcB of *E*. *coli* K-12, which was involved in fluoride resistance in a previous report [14]. As shown in Fig 1, these oral streptococci were divided into three groups based on the distribution of *eriC* and *crcB* genes: group I with only *eriC1*, group II with *eriC1* and *eriC2*, and group III with *eriC2*, *crcB1*, and *crcB2*. The *eriC1* and *crcB* genes were flanked by highly similar gene arrangements.

### Fluoride resistance of oral streptococci

**Fig 2:**
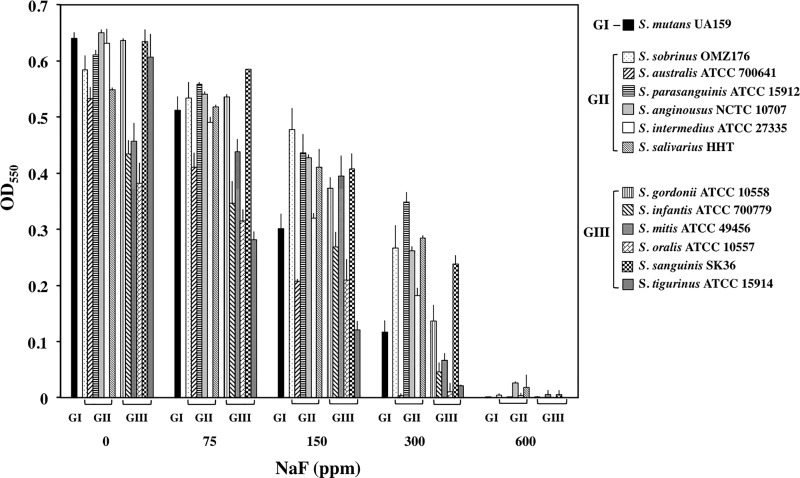
Growth yields of 13 oral streptococci at various NaF concentrations. The y-axis represents the OD_550_ after incubation for 16 h. The data represent the mean standard deviations of three independent experiments.

To examine the fluoride resistance of oral streptococci, 13 available species among the above 18 oral *Streptococcus* species were grown in BHI broth with various concentrations of NaF. These oral streptococci showed various resistances to fluoride (Fig 2). *S*. *parasanguinis*, *S*. *sanguinis*, *S*. *sobrinus*, *S*. *salivarius*, and *S*. *anginosus* grew fairly well, even in the presence of 300 ppm NaF. *S*. *australis*, *S*. *oralis*, *S*. *infantis*, *S*. *mitis*, and *S*. *tigurinus* grew poorly under the same conditions. In the presence of 600 ppm NaF, all 13 oral streptococci did not grow. Among species with the higher fluoride-resistance, *S*. *parasanguinis*, *S*. *sobrinus*, *S*. *salivarius*, and *S*. *anginosus* belong to group II and *S*. *sanguinis* belongs to group III. On the other hand, among species with the lower fluoride-resistance, *S*. *oralis*, *S*. *infantis*, *S*. *mitis*, and *S*. *tigurinus* belonged to group III and *S*. *australis* belonged to group II. *S*. *mutans*, which belonged to group I, showed medium fluoride-resistance. No strong relationship was observed between the distribution of *eriC* and *crcB* genes and fluoride resistance. Next, we explored the related gene(s) for fluoride resistance in *S*. *mutans*, *S*. *anginosus*, and *S*. *sanguinis*, which possess a fair fluoride resistance, as representative species of the three groups, respectively.

### Identification of fluoride-resistance-related genes in *S*. *mutans*

**Fig 3:**
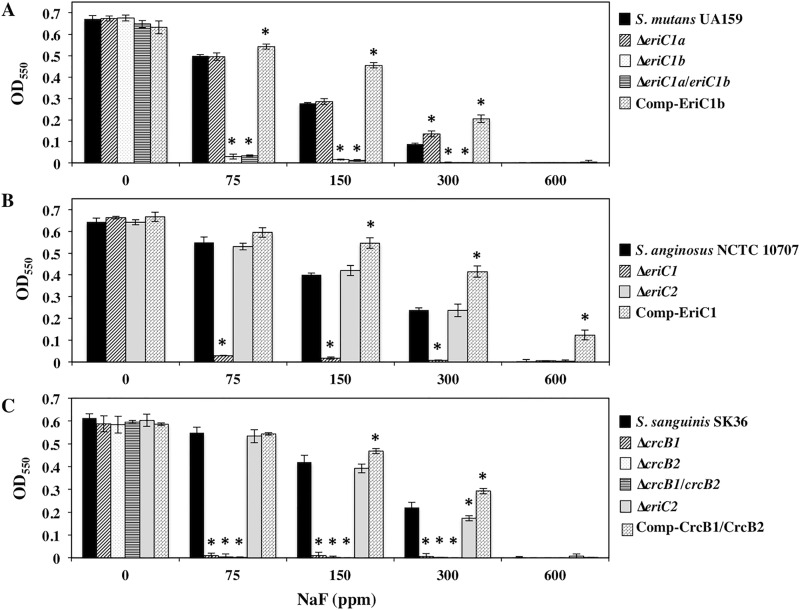
Growth yields of the parent strains, *eriC* or *crcB* deletion mutants, and complemented strains in *S. mutans* (A), *S. anginosus* (B), and *S. sanguinis* (C) at various NaF concentrations. The y-axis represents the OD_550_ after incubation for 16 h. Data represent the mean standard deviations of three independent experiments. *, significant differences against the OD_550_ value of the wild-type strain within the same NaF concentration.

Sequence homology analysis revealed two *eriC1* genes [SMU_1290 (*eriC1a*) and SMU_1289 (*eriC1b*)] in *S*. *mutans* UA159. *eriC1a* and *eriC1b* exist in tandem and in the same orientation. Both *eriC1a* and *eriC1b* encode a protein of 406 amino acids, named the chloride channel permease. The amino acid sequences deduced from *eriC1a* and *eriC1b* showed 52.6 and 52.5% similarity, respectively, to *P*. *syringae* EriC. The amino acid sequence similarity between EriC1a and EriC1b was 74.4%. To examine whether inactivation of *eriC1a* or/and *eriC1b* resulted in the loss of fluoride resistance, *eriC1a* and *eriC1b* single mutants, and the *eriC1a*/*eriC1b* double mutant were constructed. As shown in Fig 3A, the *eriC1b* mutant showed extremely limited growth in the presence of 75 ppm NaF, while it grew similarly to the wild-type UA159 in the absence of NaF. In contrast, the *eriC1a* mutant showed a growth rate similar to wild-type UA159 in both the presence and absence of NaF. Furthermore, the growth rates of the *eriC1a*/*eriC1b* double mutant and the *eriC1b* single mutant were similar in the presence of NaF. Next, gene complementation analysis was performed using the pSEP shuttle vector in the *eriC1a*/*eriC1b* double mutant. Introduction of *eriC1b* into the *eriC1a*/*eriC1b* double mutant restored fluoride resistance (Fig 3A). On the other hand, the complementation by *eriC1a* did not rescued fluoride resistance (data not shown). These results demonstrated that EriC1b was responsible for the fluoride resistance in *S*. *mutans*.

### Identification of fluoride-resistance-related genes in *S*. *anginosus*

*S*. *anginosus* possesses two types of *eriC* genes (*eriC1* and *eriC2*). *eriC1* encodes a protein of 405 amino acids with amino acid sequence similarity of 52% to the *P*. *syringae* EriC, and is named the voltage-gated chloride channel protein. *eriC2* encodes a protein of 518 amino acids with no significant amino acid sequence similarity to the *P*. *syringae* EriC. To examine whether the inactivation of *eriC1* or *eriC2* leads to the loss of fluoride resistance, the *eriC1* and *eriC2* single mutants were constructed in *S*. *anginosus* NCTC 10707. The *eriC1* mutant hardly grew in the presence of 75 ppm NaF, while the *eriC2* mutant showed a growth rate similar to that of wild-type NCTC 10707 in the presence of NaF (Fig 3B). Furthermore, complementation of *eriC1* rescued fluoride resistance (Fig 3B), while the introduction of *eriC2* did not (data not shown). These results demonstrated that EriC1 was responsible for the fluoride resistance in *S*. *anginosus*.

### Identification of fluoride-resistance-related genes in *S*. *sanguinis*

The *eriC2*, *crcB1*, and *crcB2* genes are present in *S*. *sanguinis*. The *eriC2* gene product is a protein of 518 amino acids with no significant similarity to *P*. *syringae* EriC, but with a similarity of 71% to *S*. *anginosus* EriC2, which is not involved in fluoride resistance in *S*. *anginosus*. The *crcB1* gene encodes a protein of 124 amino acids with amino acid sequence similarity of 51% to *E*. *coli* CrcB (127-aa protein), which is involved in fluoride resistance in *E*. *coli*, and the *crcB2* gene product encodes a protein of 116 amino acids with a similarity of 62.2% to *E*. *coli* CrcB. Amino acid sequence similarity between CrcB1 and CrcB2 was 58.4%. The *crcB1* and *crcB2* genes exist in tandem and in the same orientation. To examine whether the inactivation of *eriC2*, *crcB1*, or *crcB2* leads to the loss of fluoride resistance, *eriC2*, *crcB1*, and *crcB2* single mutants were constructed in *S*. *sanguinis* SK36. Inactivation of *eriC2* did not affect fluoride resistance of wild-type SK36 (Fig 3C). On the other hand, both *crcB1* and *crcB2* single mutants showed extremely limited growth in the presence of 75 ppm NaF, while they grew similarly to wild-type SK36 in the absence of NaF (Fig 3C). The *crcB1*/*crcB2* double mutant showed a growth rate similar to that of the *crcB1* or *crcB2* single mutants in both the absence and presence of NaF (Fig 3C). Complementation of *crcB1/crcB2* in the *crcB1/crcB2* double mutant restored fluoride resistance to wild-type levels (Fig 3C), while the introduction of *eriC2* did not (data not shown). Furthermore, the *crcB1/crcB2* mutant complemented by either *crcB1* or *crcB2* also restored fluoride resistance, while the fluoride resistance of these strains did not reach the level of wild type strain (data not shown). These results demonstrated that both *crcB1* and *crcB2* genes were critical for fluoride resistance in *S*. *sanguinis*.

### Complementation of the fluoride-resistance-related genes between *S*. *mutans* and *S*. *anginosus* or *S*. *mutans* and *S*. *sanguinis*

**Fig 4:**
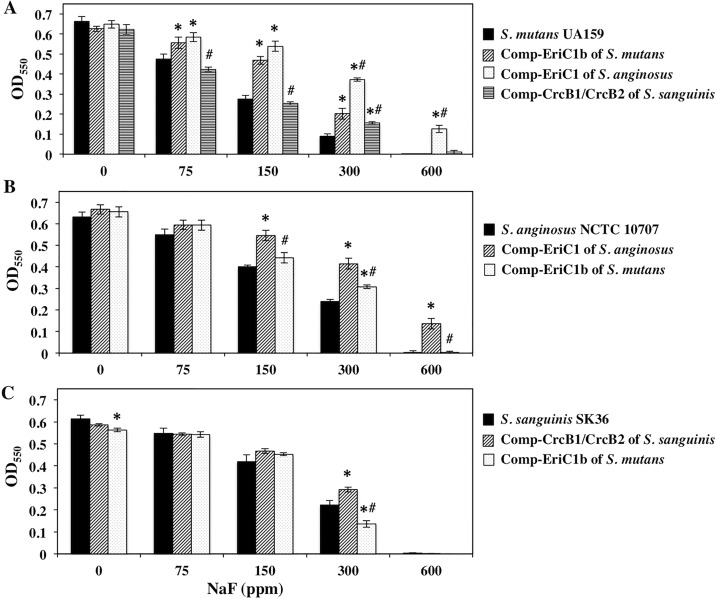
Growth yields of the parent strains and the complemented strains. (A) Complementation by *S*. *mutans* EriC1b, *S*. *anginosus* EriC1, or *S*. *sanguinis* CrcB1/CrcB2 in the *S*. *mutans* EriC1a/EriC1b double mutant. (B) Complementation by *S*. *anginosus* EriC1 or *S*. *mutans* EriC1b in the *S*. *anginosus* EriC1 mutant. (C) Complementation by *S*. *sanguinis* CrcB1/CrcB2 or *S*. *mutans* EriC1b in the *S*. *sanguinis* CrcB1/CrcB2 double mutant. The y-axis represents the OD_550_ after incubation for 16 h. The data represent the mean standard deviations of three independent experiments. *, significant differences against the OD_550_ value of the wild-type strain within the same NaF concentration; ^**#**^, significant differences against the OD_550_ value of the strain complemented by its own fluoride-resistance gene(s) within the same NaF concentration.

We explored whether fluoride-resistance-related genes were able to complement each other between *S*. *mutans* and *S*. *anginosus* or *S*. *mutans* and *S*. *sanguinis*. As shown in Fig 4A, complementation of *eriC1a*/*eriC1b* by *S*. *anginosus eriC1* in the *eriC1a*/*eriC1b* double mutant rescued fluoride resistance, and the fluoride resistance ability of this complemented strain was higher than that complemented by its own *eriC1b*. Complementation of *eriC1* by *eriC1b* in *S*. *anginosus* also restored fluoride resistance, and the fluoride resistance ability of this transformant was lower than that complemented by its own *eriC1* (Fig 4B). Next, we performed complementation of *eriC* and *crcB* between *S*. *mutans* and *S*. *sanguinis*. Both the *eriC1a*/*eriC1b* mutant complemented by *crcB1*/*crcB2* and the *crcB1*/*crcB2* mutant complemented by *eriC1b* showed almost restored fluoride resistance (Fig 4A and 4C), as well as that *E*. *coli crcB* knock-out mutant rescued fluoride resistance by introduction of the fluoride-resistance-related EriCs of other bacteria [21]. The fluoride resistance ability of the *eriC1a*/*eriC1b* mutant complemented by *crcB1*/*crcB2* was lower than that complemented by its own *eriC1b*, while the fluoride resistance ability of *S*. *sanguinis* wild-type SK36 tended to be higher than that of *S*. *mutans* wild-type UA159. Furthermore, we predicted that the co-existence of different F^−^ channels (EriC and CrcB) may cause the additive effect on fluoride resistance in oral *Streptococcus* species. It was examined whether introductions of *eriC1b* and *crcB1*/*crcB2* into wild-type *S*. *sanguinis* and *S*. *mutans*, respectively, affects fluoride resistance in the wild-type strains. Neither introduction of *eriC1b* nor *crcB1*/*crcB2* increased the fluoride resistance of the wild-type strain (data not shown).

## Discussion

ClC-type anion-selective channels are widespread throughout eukaryotic organisms and play a crucial role in controlling the ionic composition of the cytoplasm and the volume of cells, as well as the regulation of membrane electrical excitability. Although these channels may conduct other anions (e.g., I^−^ or NO_3_^−^) better than Cl^−^, they are often called chloride channels because Cl^−^ is the most abundant anion in organisms [22]. BLAST homology searches revealed that many microbial genomes contain members of the ClC family, and the prokaryotic ClC channel was designated an *E*. *coli*-derived ClC chloride channel homolog (EriC) [23]. *E*. *coli* EriC has been confirmed experimentally to function as a ClC Cl^−^ channel. On the other hand, *P*. *syringae* EriC has been reported to be associated with fluoride resistance [14]. The *crcB* (confers resistance to camphor B) gene has previously been implicated in resistance to camphor-induced chromosome decondensation. However, it has recently been reported that an *E*. *coli crcB* knock-out mutant became sensitive to fluoride, and *crcB* is predicted to code for membrane proteins belonging to a superfamily composed predominantly of transporters [14]. Furthermore, it was shown that the eukaryotic *crcB* homolog [renamed *FEX* (fluoride exporter)] encodes a previously unrecognized class of fluoride exporters required for survival under standard environmental conditions [24].

Oral streptococci are classified into four groups; namely, the mutans, the salivarius, the mitis, and the anginosus groups, according to their 16S rRNA sequences [25]. The present study suggests that oral streptococci possess either *eriC1* or *crcB*, which are associated with fluoride resistance. Among oral streptococci examined in this study, all *Streptococcus* species belonging to the mutans and the salivarius groups contained EriC1. Nine of 11 *Streptococcus* species belonging to the mitis group possessed CrcB and the other 2 species possessed EriC1. Although a distinct relationship between the type of genes (*eriC1* or *crcB*) and the fluoride-resistance level was not observed, oral streptococci with *eriC1* were prone to higher fluoride resistance than those with *crcB*. The difference in the fluoride-resistance level related to the gene types suggests that the mechanisms through EriC1 and CrcB are not identical.

Baker *et al*. [14] showed that *P*. *syringae* EriC and *C*. *difficile* EriC associated with fluoride resistance commonly carry a specific set of amino acids in the conserved anion selectivity filter region [26], which are distinct from validated chloride-specific EriC proteins (Table 1). In this study, two novel genes encoding EriC1b of *S*. *mutans* and EriC1 of *S*. *anginosus* were shown to be involved in fluoride-resistance, and subsequently seven EriC1s in other oral streptococci were predicted to be responsible for fluoride resistance of these organisms based on amino acid sequence homology. Our table shows amino acid sequences of the conserved anion selectivity filter region of the 18 EriC homologs; 3 EriCs are Cl^−^ channels and 15 EriCs are predicted to be F^−^ channels. All EriC1s of oral streptococci possessed a similar set of amino acid sequences to those of the F^−^ channel EriCs, but they were distinct from those of Cl^−^ channel EriCs. Although it is possible that EriC1s in oral streptococci may be generally responsible for the fluoride resistance, the role of EriC1s from oral streptococci other than *S*. *mutans* and *S*. *anginosus* in fluoride resistance should be examined. The amino acid sequences of the region in the streptococci were more similar to those of *C*. *difficile*, *Eubacterium ventriosum*, and *Lactococcus lactis* EriCs than those of *P*. *syringae*, *Pirellula staleyi*, and *Ralstonia picketti* EriCs, possibly due to the difference between Gram-positive and Gram-negative bacteria. On the other hand, EriC2s identified in all oral streptococci examined in this study, except for *S*. *mutans*, possessed amino acid sequences with conserved Cl^−^ channels (data not shown), suggesting that EriC2 may be related to Cl^−^ channels.

**Table 1:** Amino acid sequences of the conserved anion selectivity filter region of several EriCs. Eight EriCs (dotted box) were shown to be involved in fluoride resistance [14, 21]. Solid boxes show the amino acid sequences associated with fluoride resistance. Related EriCs commonly contain a specific set of amino acids, which are distinct from validated chloride-specific EriCs.

Substrate	Organism	Cl^−^ selectivity filter residues
Cl^−^	human (ClC-1)	**GSGIP……GKEGP……GGFMP……Y**
Cl^−^	*Escherichia coli*	**GSGIP……GREGP……GIFAP……Y**
Cl^−^	*Salmonella typhimurium*	**GSGIP……GREGP……GIFAP……Y**
F^−^	*Pseudomonas syringae*	**GNNLI……GREGT……GEVTP……Y**
F^−^	*Pirellula staleyi*	**GNNLL……GREGT……GEVTP……Y**
F^−^	*Ralstonia picketti*	**GNNLL……GREGT……GEVTP……Y**
F^−^	*Clostridium difficile*	**GMNLI……GREGV……GEVTP……Y**
F^−^	*Eubacterium ventriosum*	**GMNLV……GREGV……GEVTP……Y**
F^−^	*Lactococcus lactis*	**GMGLI……GREGV……GEVTP……Y**
F^−^	*Streptococcus mutans*	**GMGLI……GREGV……GEVTP……Y**
F^−^	*Streptococcus anginosus*	**GMTLI……GREGV……GEVTP……Y**
F^−^	*Streptococcus sobrinus*	**GMGLV……GREGV……GEVTP……Y**
F^−^	*Streptococcus australis*	**GMELL……GREGV……GEVTP……Y**
F^−^	*Streptococcus parasanguinis*	**GMGLI……GREGV……GEVTP……Y**
F^−^	*Streptococcus salivarius*	**GMGLI……GREGV……GEVTP……Y**
F^−^	*Streptococcus vestibularis*	**GMGLI……GREGV……GEVTP……Y**
F^−^	*Streptococcus constellatus*	**GMTLI……GREGV……GEVTP……Y**
F^−^	*Streptococcus intermedius*	**GMTLI……GREGV……GEVTP……Y**

Liao *et al*. [15] have reported that 2 SNPs related to fluoride resistance were identified in the genome of the fluoride resistant strain *S*. *mutans* C180-2FR. These were located in the region of the gene cluster composed of SMU_1291, *eriC1a*, and *eriC1b*; one in its promoter region and the other in *eriC1b*. The expression of the cluster comprised of three genes was approximately 10-fold higher in C180-2FR than in the parent strain C180-2. Murata and Hanada [27] have shown that both *eriC1a* and *eriC1b* were involved in the fluoride resistance in *S*. *mutans*. We demonstrated that *eriC1b* was involved in the fluoride resistance of *S*. *mutans*, but deletion of *eriC1a* did not affect the fluoride resistance. Contribution of EriC1a in fluoride resistance of *S*. *mutans* in the previous study [27] was inconsistent with our result. We are not able to explain such a difference between these two studies. The discrepancy might be caused by different cultural conditions: that is, the previous study utilized anaerobic condition, while the present study did aerobic condition with 5% CO_2_. It is unknown why *S*. *mutans* possesses two EriC1s unlike other oral streptococci and also why inactivation of *eriC1a* does not result in loss of fluoride resistance even though EriC1a and EriC1b showed amino acid sequence similarity of 74% that is higher than the similarity of 52.5% between EriC1b and *P*. *syringae* EriC1 actually contributing to fluoride resistance. However, higher homology of total amino acid sequence does not necessarily guarantee the function of protein.

In the present study, we demonstrated that both *crcB1* and *crcB2* genes were critical for fluoride resistance in *S*. *sanguinis*, although a single *crcB* is responsible for the fluoride resistance in *E*. *coli*. In addition, all oral streptococci classified in group III in accordance with genes associated with anion channels possessed both *crcB1* and *crcB2*. Using the KEGG (Kyoto Encyclopedia of Genes and Genomes) Organisms database (http://www.genome.jp/kegg/catalog/org_list.html), we found that *Bacillus subtilis* and *Staphylococcus aureus*, Gram-positive bacteria, possess two types of *crcB*s, while only a single *crcB* gene was found in genomes of *E*. *coli* and *Salmonella enterica*, Gram-negative bacteria. CrcBs of *E*. *coli* was characterized to be a fluoride efflux channel [14]. However, the function of CrcBs in the other bacteria has not yet well elucidated. Although, in this study, we aimed to broadly identify genes involved in the fluoride resistance in oral streptococci, the reason why two *crcB*s exist in Gram-positive bacteria in contrast to Gram-negative bacteria, in addition to a unique genotype composed of two genes encoding EriC1 in *S*. *mutans*, would be a promising research target in future.

Complementation between *S*. *mutans* EriC1b and *S*. *anginosus* EriC1 and that between *S*. *mutans* EriC1b and *S*. *sanguinis* CrcB1/CrcB2 were confirmed in this study. Introduction of *S*. *anginosus* EriC1 into an EriC1s-knockout mutant of *S*. *mutans* increased fluoride resistance when compared with the same complementation experiment with EriC1b. On the other hand, when EriC1b was transferred into the EriC1-knockout mutant of *S*. *anginosus*, fluoride resistance of the complemented strain was lower than that complemented with its own EriC1. The fluoride resistance level of *S*. *anginosus* NCTC10707 was higher than that of *S*. *mutans* UA159, and the differences in fluoride resistance levels between the above species may be attributed to differences in the kinetics of ion transport between EriC1s. Moreover, both the EriC1s-knockout mutant of *S*. *mutans* complemented by *S*. *sanguinis* CrcB1/CrcB2 and the CrcB1/CrcB2-knockout mutant complemented by *S*. *mutans* EriCb showed restored fluoride resistance. However, neither introduction of *S*. *sanguinis* CrcB1/CrcB2 into wild-type *S*. *mutans* nor *S*. *mutans* EriC1b into wild-type *S*. *sanguinis* increased the fluoride resistance level of the wild-type strain. Co-existence of different F^−^ channels (EriC and CrcB) did not cause the additive effect on fluoride resistance in oral *Streptococcus* species.

Fluoride application is highly effective in preventing dental caries. On the other hand, we must consider that fluoride has anti-bacterial effects. The frequent application of fluoride results in the emergence of fluoride resistant strains, leading to the dysbiosis of oral microflora. This disturbance may affect not only oral health conditions, but also general health conditions. Exploring the mechanism of fluoride resistance may contribute to countermeasures against the risk of emergence of the fluoride-resistant strains in oral microflora.
